# Supplementary material for: A rice QTL GS3.1 regulates grain size through metabolic-flux distribution between flavonoid and lignin metabolons without affecting stress tolerance
Source: Commun Biol. 2021 Oct 7;4:1171. doi: 10.1038/s42003-021-02686-x (PMC8497587; doi:10.1038/s42003-021-02686-x)
Supplement: Supplementary file 2 — Supplementary information [file 42003_2021_2686_MOESM2_ESM.pdf]

Supplementary information

A rice QTL *GS3.1* regulates grain size through metabolic-flux distribution between flavonoid and lignin metabolons without affecting stress tolerance

Yi-Min Zhang[1,2], Hong-Xiao Yu[1,2], Wang-Wei Ye[1], Jun-Xiang Shan[1], Nai-Qian Dong[1], Tao Guo[1], Yi Kan[1,2], You-Huang Xiang[1,2], Hai Zhang[1,3], Yi-Bing Yang[1,2], Ya-Chao Li[1,3], Huai-Yu Zhao[1,2], Zi-Qi Lu[1,3], Shuang-Qin Guo[1,2], Jie-Jie Lei[1,2], Ben Liao[1,3], Xiao-Rui Mu[1,2], Ying-Jie Cao[1,2], Jia-Jun Yu[1,3], Hong-Xuan Lin[1,2,3]*

[1]National Key Laboratory of Plant Molecular Genetics, CAS Centre for Excellence in Molecular Plant Sciences and Collaborative Innovation Center of Genetics & Development, Shanghai Institute of Plant Physiology and Ecology, Chinese Academy of Sciences, Shanghai 200032, China

[2]University of the Chinese Academy of Sciences, Beijing 100049, China

[3]School of Life Science and Technology, ShanghaiTech University, Shanghai 201210, China

*Correspondence: hxlin@cemps.ac.cn or hxlin@sibs.ac.cn

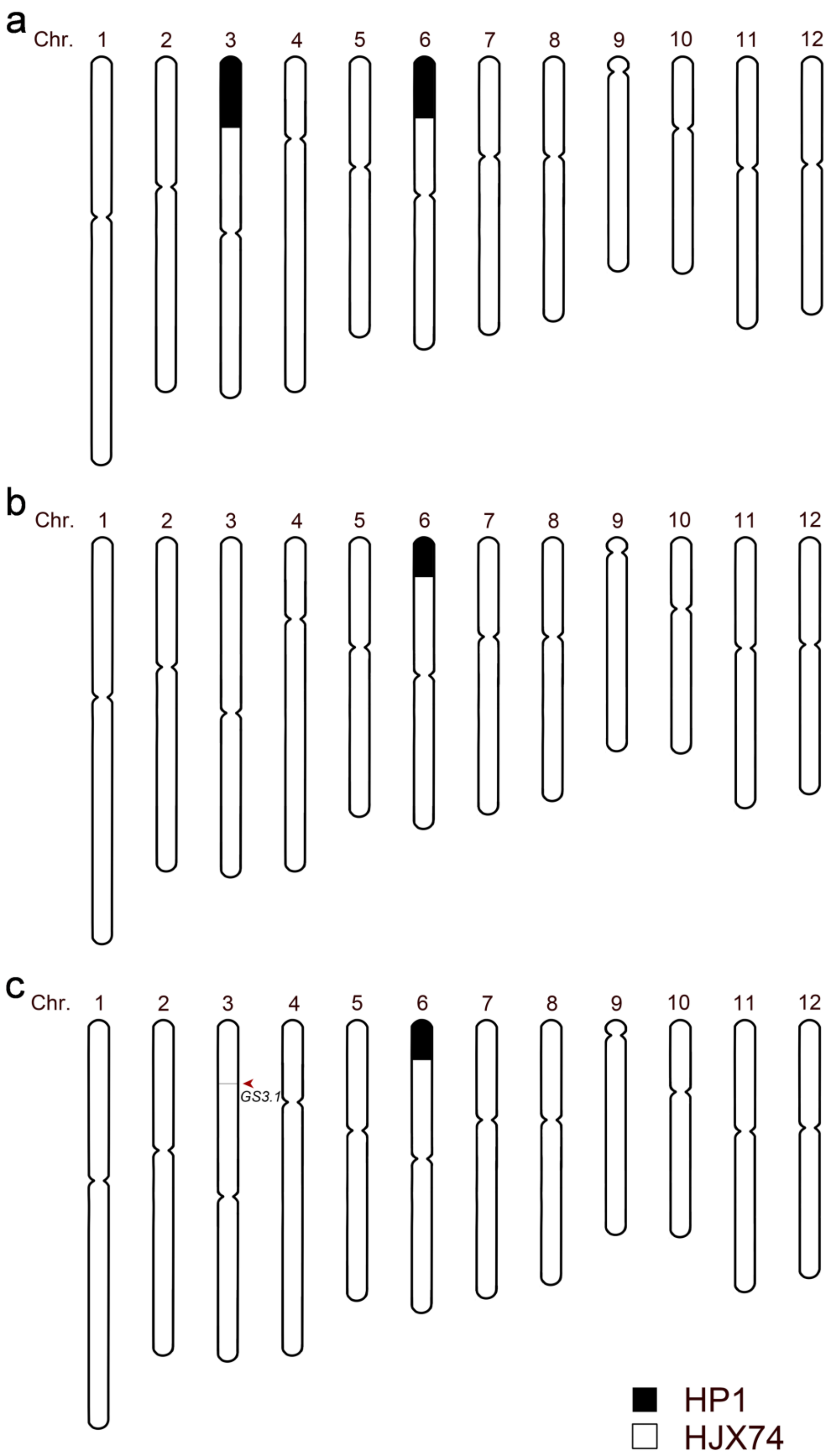

Supplementary Figure 1. Cytogenetic image of genetic materials of *GS3.1*. The cytogenetic image of the CSSL containing *GS3.1* (**a**), NIL-*GS3.1*^{HJX} (**b**) and NIL-*GS3.1*^{HP} (**c**).

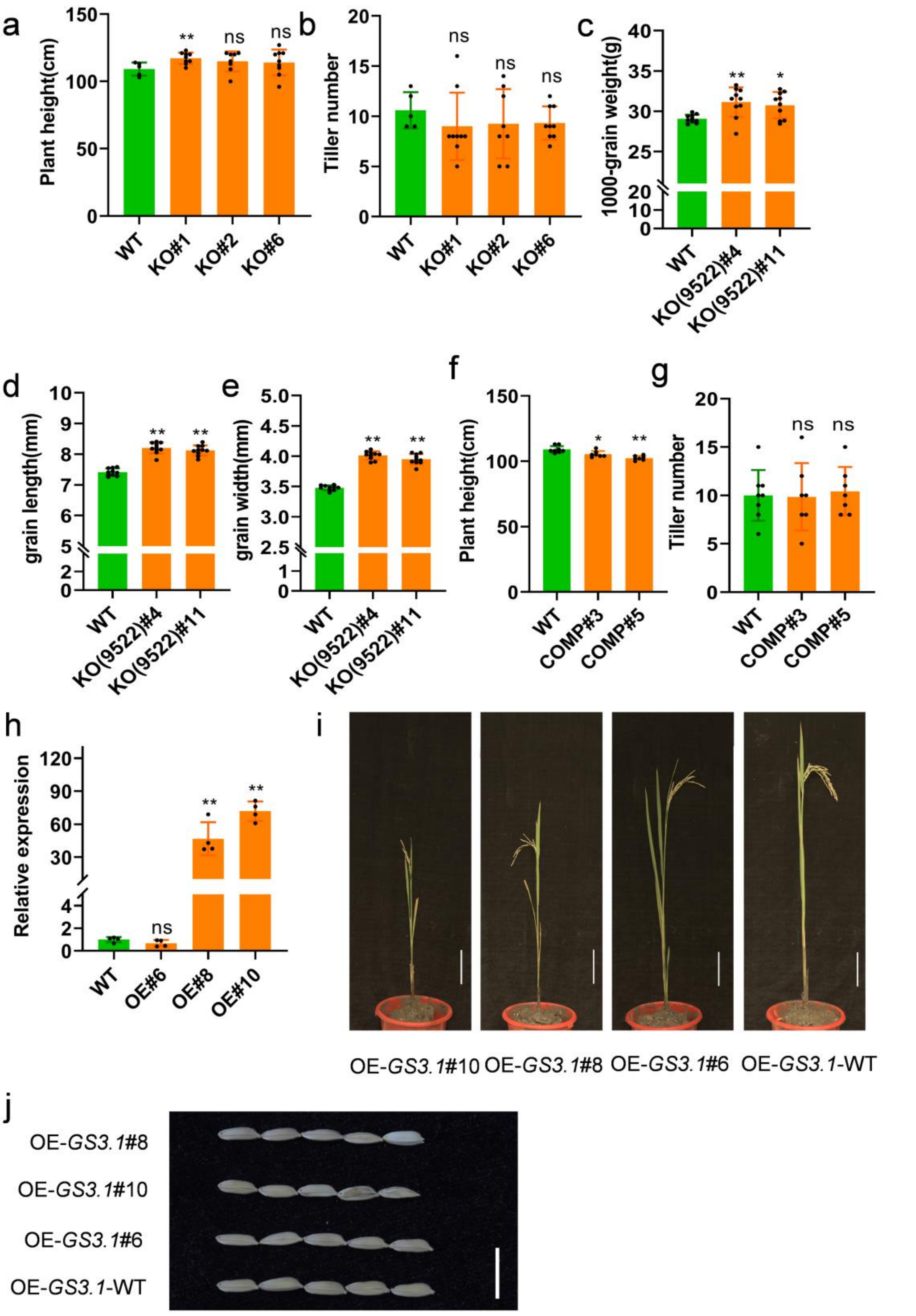

Supplementary Figure 2. Phenotypes of *GS3.1* transgenic lines. Comparison of plant height (**a**) and tiller number (**b**) between KO-*GS3.1* transgenic lines KO-*GS3.1*#1, KO-*GS3.1*#2, KO-*GS3.1*#6, and the wild-type control (n ≥ 5 plants). Comparison of 1000-grain weight (**c**), grain length (**d**) and grain width (**e**) between *GS3.1* knock-out lines in the 9522 background KO-*GS3.1*(9522)-4, KO-

GS3.1(9522)-11, and the 9522 control (n = 10 plants). Comparison of plant height (**f**) and tiller number (**g**) between *GS3.1* complementation lines COMP-*GS3.1* #3, COMP-*GS3.1* #5, and the NIL-*GS3.1*^{HP} control (n ≥ 7 plants). **h**, The relative expression levels of *GS3.1* in *GS3.1* overexpression lines OE-*GS3.1*-6, OE-*GS3.1*-8, OE-*GS3.1*-10, and the wild-type control leaves determined by qRT-PCR (n = 4 biological replicates). The actin gene was used for normalization. Plant architecture (**i**) and mature grains (**j**) of *GS3.1* overexpression lines OE-*GS3.1*-6, OE-*GS3.1*-8, OE-*GS3.1*-10, and the wild-type control. Scale bar = 10 cm (**i**) and 1cm (**j**). The values in **a-h** represent the mean ± s.d.. **P* < 0.05 and ***P* < 0.01 indicate significant differences compared to the control in two-tailed Student's *t*-tests. The source data underlying Supplementary Figure 2a-h are provided as Source Data file.

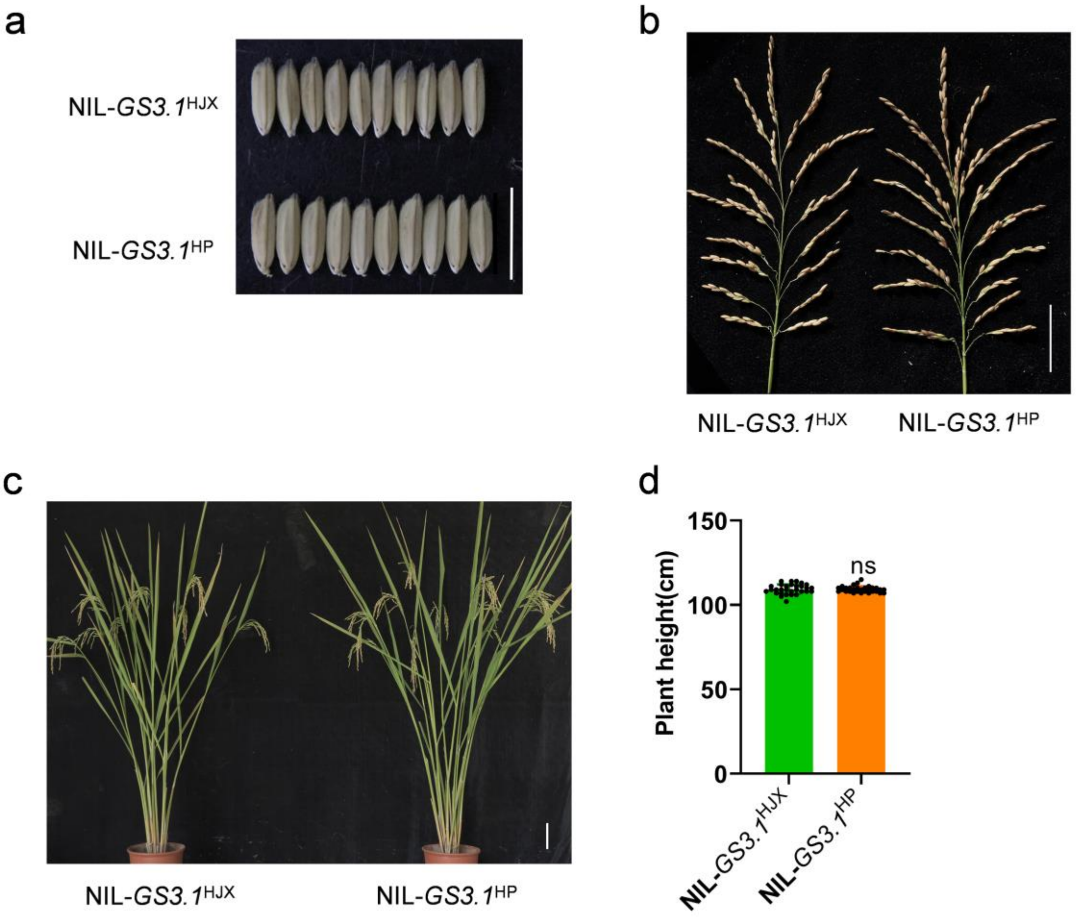

Supplementary Figure 3. Phenotypes of NIL-*GS3.1*^{HP} and NIL-*GS3.1*^{HJX}. Mature grains in the grain width direction (**a**), main panicle (**b**) and plant architecture (**c**) of NIL-*GS3.1*^{HP} and NIL-*GS3.1*^{HJX}. Scale bar =1 cm (**a**), 5 cm (**b**) and 10 cm (**c**). **d**, Comparison of plant height of NIL-*GS3.1*^{HP} and NIL-*GS3.1*^{HJX} (n = 30 plants). The values in **d** represent the mean ± s.d.. *$P < 0.05$ and **$P < 0.01$ indicate significant differences compared to NIL-*GS3.1*^{HJX} in two-tailed Student's *t*-tests. The source data underlying Supplementary Figure 3d are provided as Source Data file.

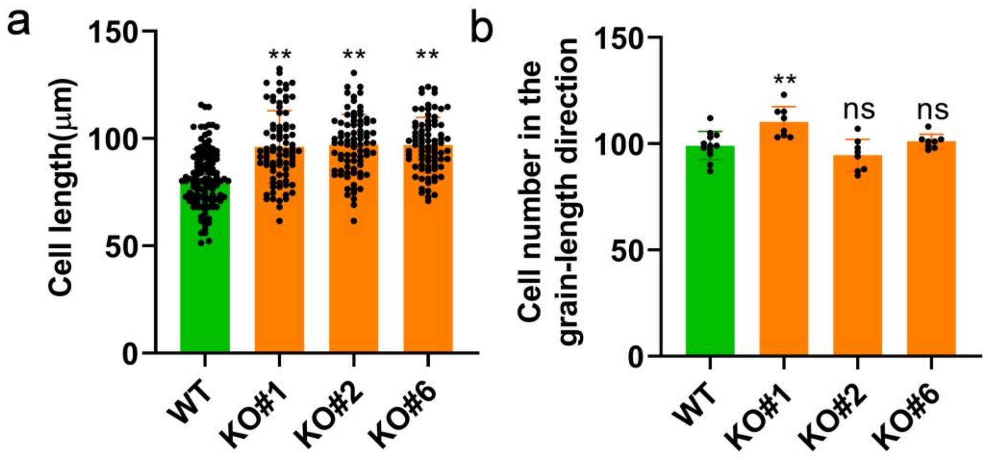

Supplementary Figure 4. Length and number of spikelet hull cells of KO-*GS3.1* and the wild-type control. Comparison of the outer epidermal cell length (**a**) and cell number in the grain-length direction (**b**) between *GS3.1* knock-out lines, KO-*GS3.1*, and the wild-type control (n= 8 spikelet hulls of KO-*GS3.1* lines and 12 spikelet hulls of wild-type control, 10 cells per spikelet hull for cell length measuring). The values in **a** and **b** represent the mean ± s.d.. *P < 0.05 and **P < 0.01 indicate significant differences compared to the wild-type control in two-tailed Student's *t*-tests. The source data underlying Supplementary Figure 4a and b are provided as Source Data file.

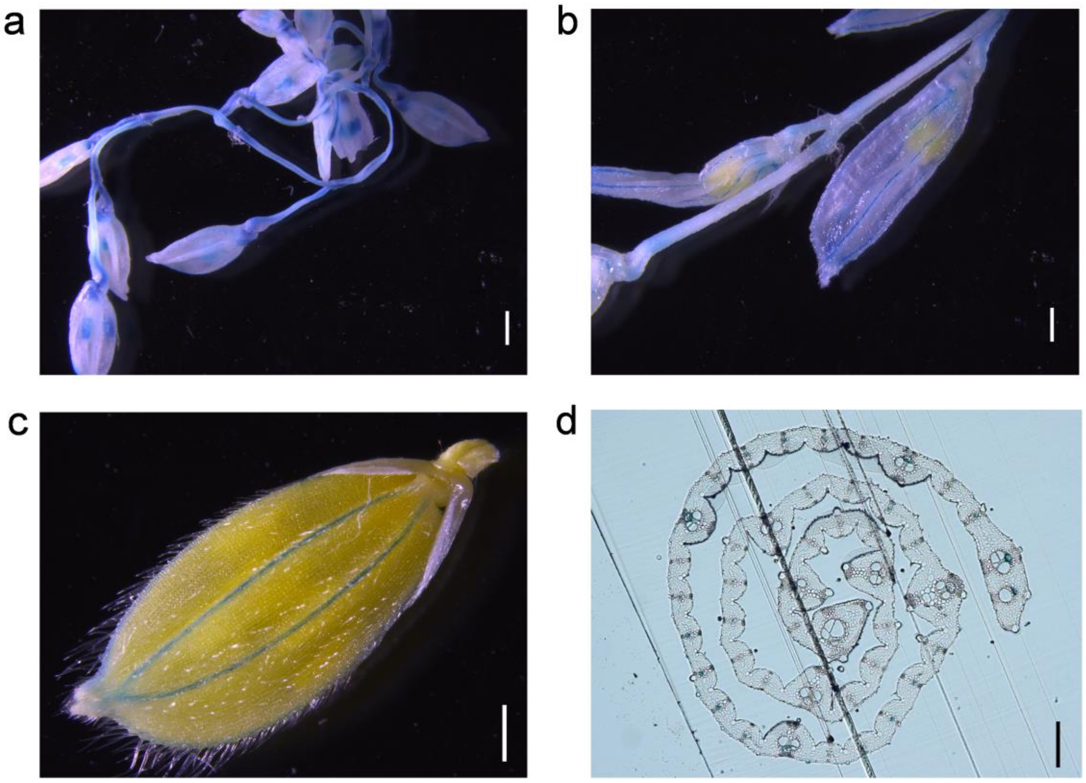

Supplementary Figure 5. GUS staining of *Pro GS3.1*^{HJX}*:GUS* transgenic lines. The GUS staining of *Pro GS3.1*^{HJX}*:GUS* of 8 cm young panicle (**a**), 15 cm young panicle (**b**) and grain before heading(**c**). Scale bar =1 mm. **d**, Micrographs of a cross-section of a young leaf. Scale bar =50 μm.

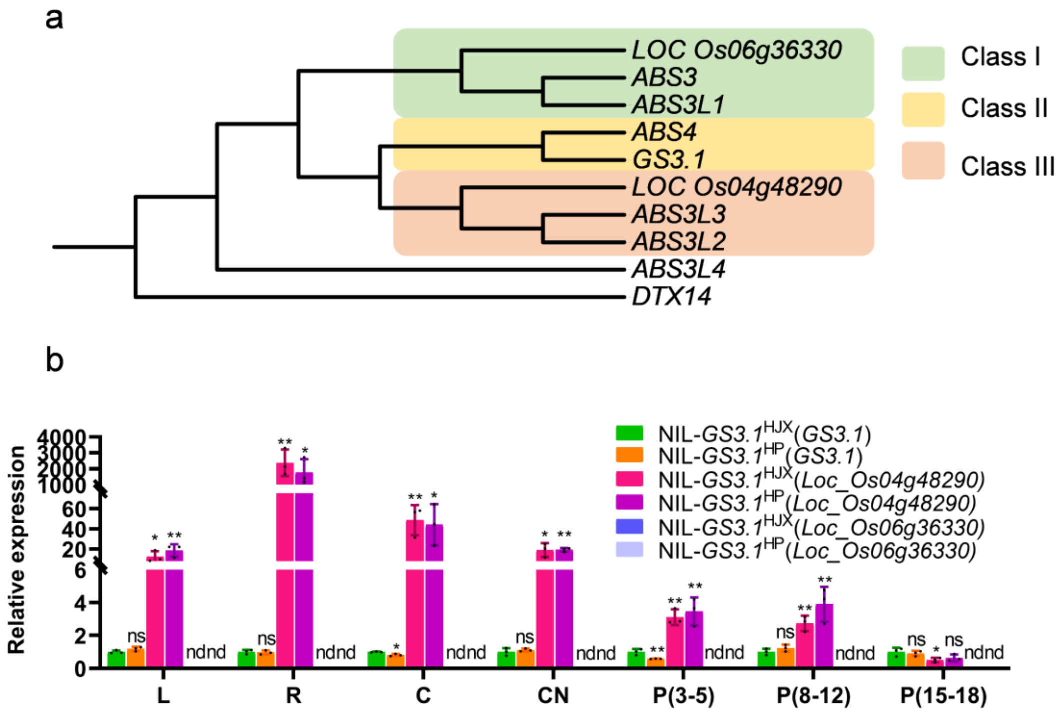

Supplementary Figure 6. The expression pattern relationship between GS3.1 and its redundant paralogs. a, The phylogenetic tree constructed by the maximum-likelihood method showed the phylogenetic relationship between genes of the *GS3.1* subfamily in rice and Arabidopsis. Four phylogenetic clades were observed, and of which each clade contains a rice gene except *ABS3L4* clade. **b**, The relative expression levels of *GS3.1* and its paralog in NIL-*GS3.1*^{HJX} and NIL-*GS3.1*^{HP} leaves (L), roots (R), culm nodes (CN), culms (C) and panicles (P, numbers indicate the length of young panicles, cm) as determined by qRT-PCR (n = 3 biological replicates). The actin gene was used for normalization. The values in **b** represent the mean ± s.d.. *$P < 0.05$ and **$P < 0.01$ indicate significant differences compared to NIL-*GS3.1*^{HJX} (*GS3.1*) in two-tailed Student's *t*-tests. The symbol nd means no detected. The source data underlying Supplementary Figure 6b are provided as Source Data file.

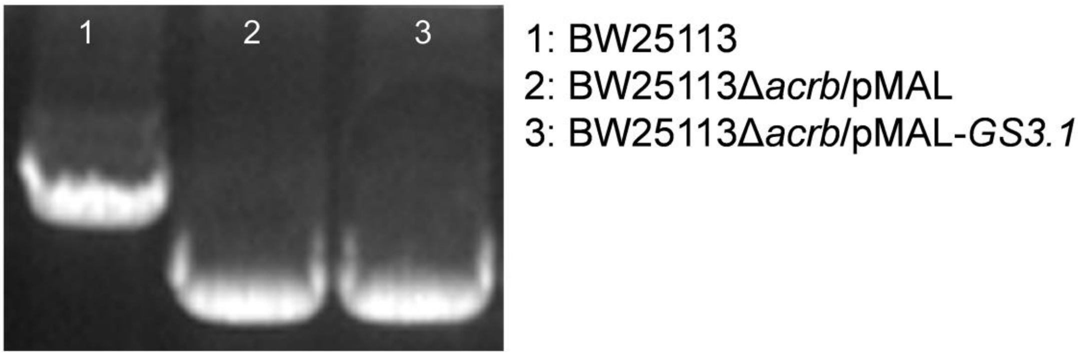

Supplementary Figure 7. Genotypes of strains in growth complementation assay. Electrophoresis bands of *acrb* amplified by PCR. The band with a large molecular weight showed the wild-type *acrb* gene (line 1) and the bands with a smaller molecular weight showed the mutant *acrb* gene (line 2 and 3).

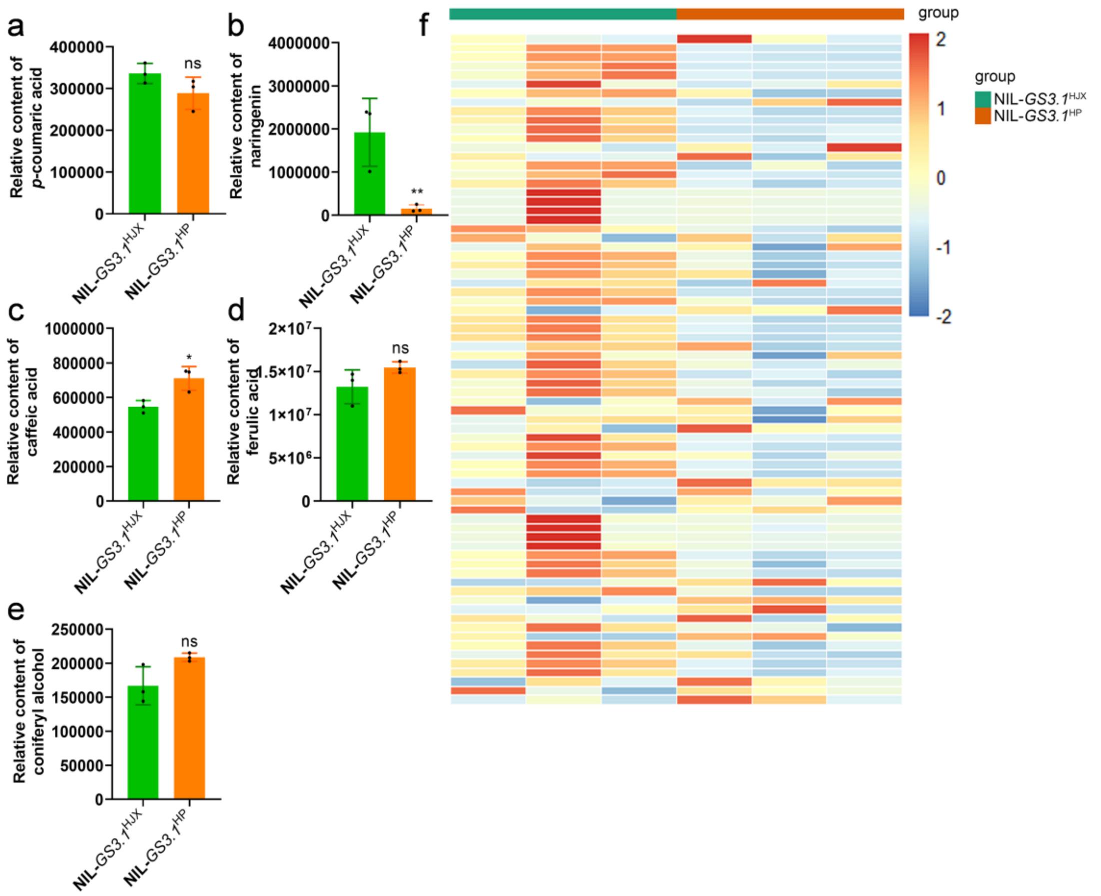

Supplementary Figure 8. Comparison of flavonoid and lignan contents between NIL-*GS3.1*^{HJX} and NIL-*GS3.1*^{HP}. Comparison of the relative content of *p*-coumaric acid (**a**), naringenin (**b**), caffeic acid (**c**)**,** ferulic acid (**d**) and coniferyl alcohol (**e**) in NIL-*GS3.1*^{HJX} panicles and NIL-*GS3.1*^{HP} panicles (n = 3 biological replicates, 4 plants per replicates). **f**, Heat maps of the relative content of flavonoids in NIL-*GS3.1*^{HJX} and NIL-*GS3.1*^{HP} determined using metabolomics data. Standard-scores (Z-scores) were used as the numerical metrics to evaluate the standard deviations from the mean of the corresponding samples. The values in **a**–**e** represent the mean ± s.d.. *$P < 0.05$ and **$P < 0.01$ indicate significant differences compared to NIL-*GS3.1*^{HJX} in two-tailed Student's *t*-tests. The source data underlying Supplementary Figure 8a-f are provided as Source Data file.

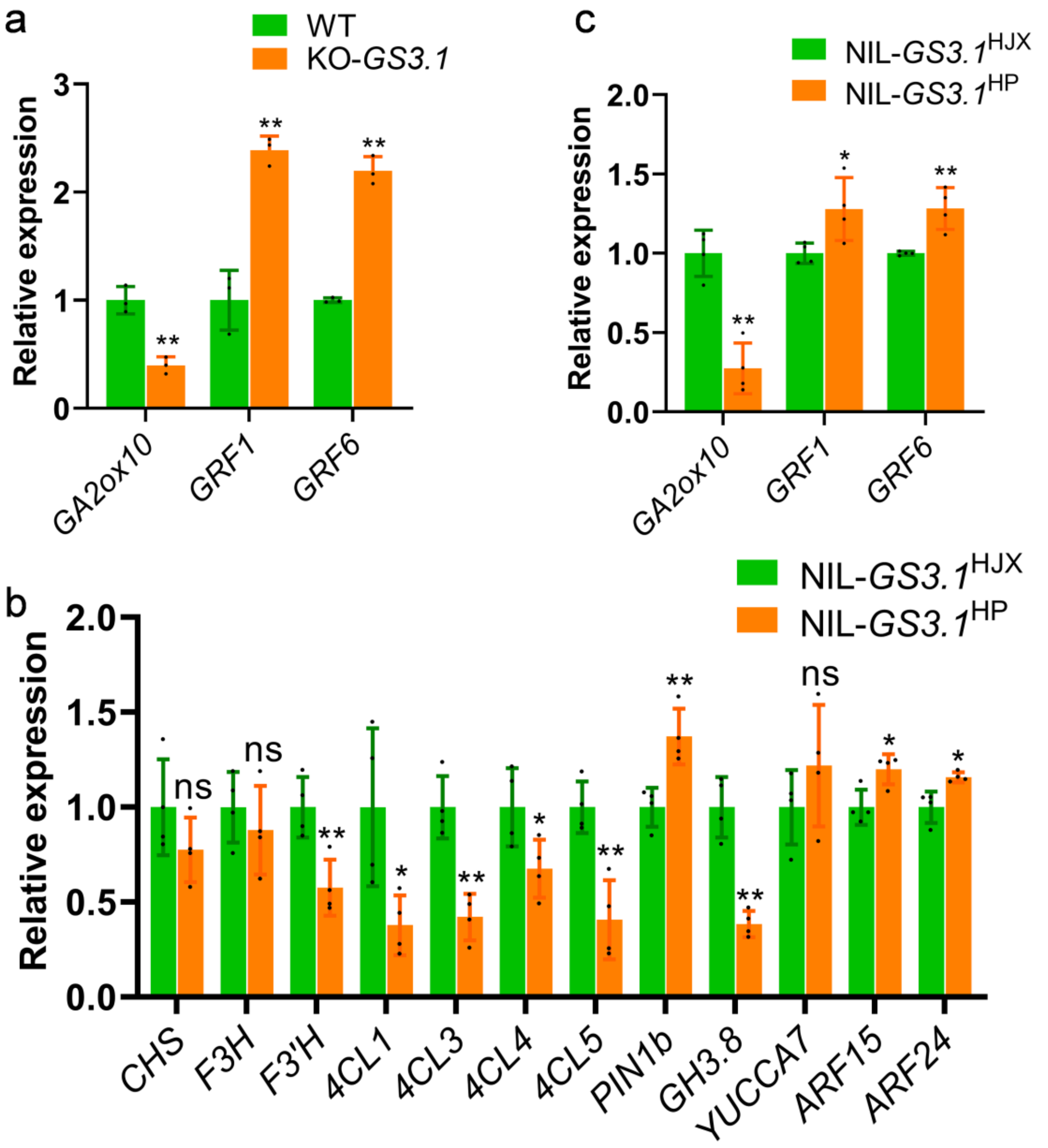

Supplementary Figure 9. The relative expression level of marker-genes. a, The relative expression of GA-related genes in KO-*GS3.1* panicles and wild-type control panicles (n = 3 biological replicates). **b**, The relative expression of flavonoid and lignan biosynthetic genes and auxin-related genes in NIL-*GS3.1*^{HJX} panicles and NIL-*GS3.1*^{HP} panicles (n = 4 biological replicates). **c**, The relative expression of GA-related genes in NIL-*GS3.1*^{HJX} panicles and NIL-*GS3.1*^{HP} panicles (n = 4 biological replicates). The values in **a** represent the mean ± s.d.. $*P < 0.05$ and $**P < 0.01$ indicate significant differences compared to the wild-type control in two-tailed Student's *t*-tests. The values in **b** and **c** represent the mean ± s.d.. $*P < 0.05$ and $**P < 0.01$ indicate significant differences compared to NIL-*GS3.1*^{HJX} in two-tailed Student's *t*-tests. The source data underlying Supplementary Figure 9a-c are provided as Source Data file.

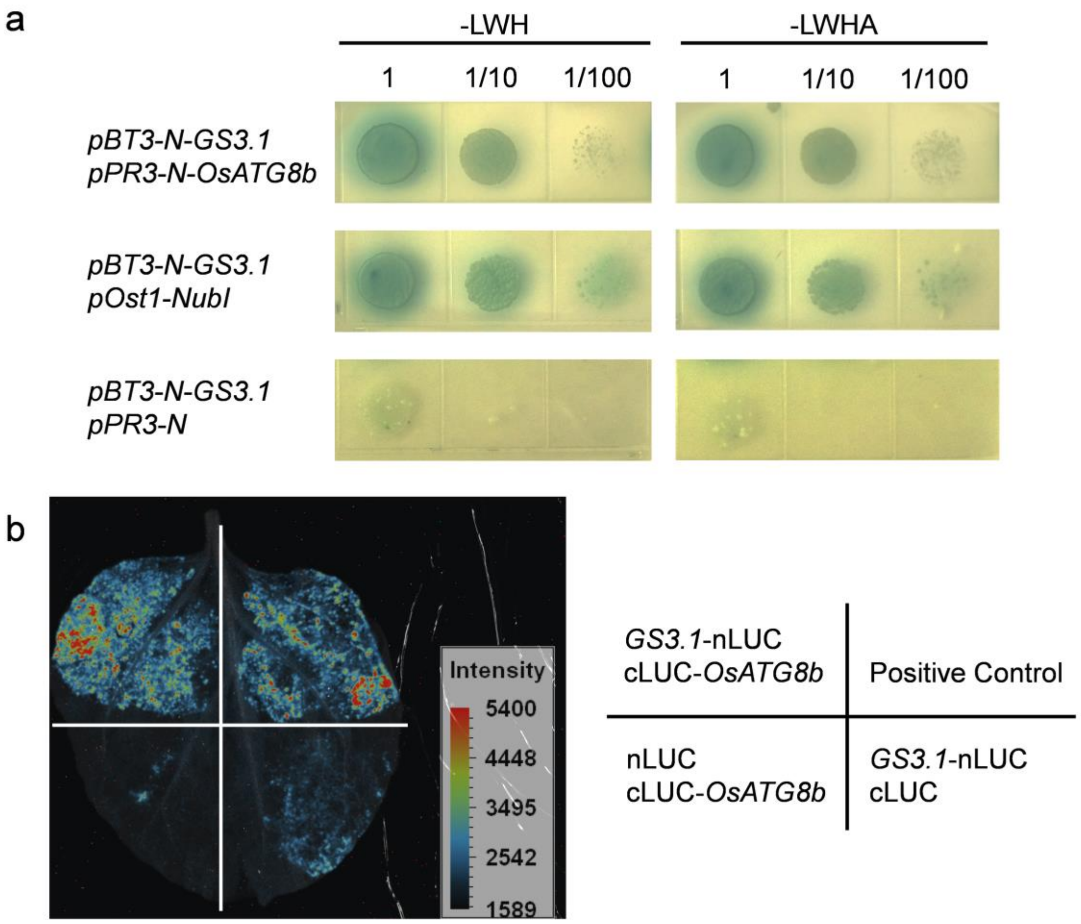

Supplementary Figure 10. The interaction between *GS3.1* and *OsATG8b*. a, Interaction between *GS3.1* and *OsATG8b* in a split-ubiquitin assay. pOst1-NubI was used as a positive control prey and empty pPR3-N was used as a negative control prey. The growth of yeast colonies on dropout medium supplemented with X-gal (-LWH+X-α-gal and –LWHA+X-α-gal). **b**, Interaction between *GS3.1* and *OsATG8b* in a luciferase complementation assay in *N. tabacum*.

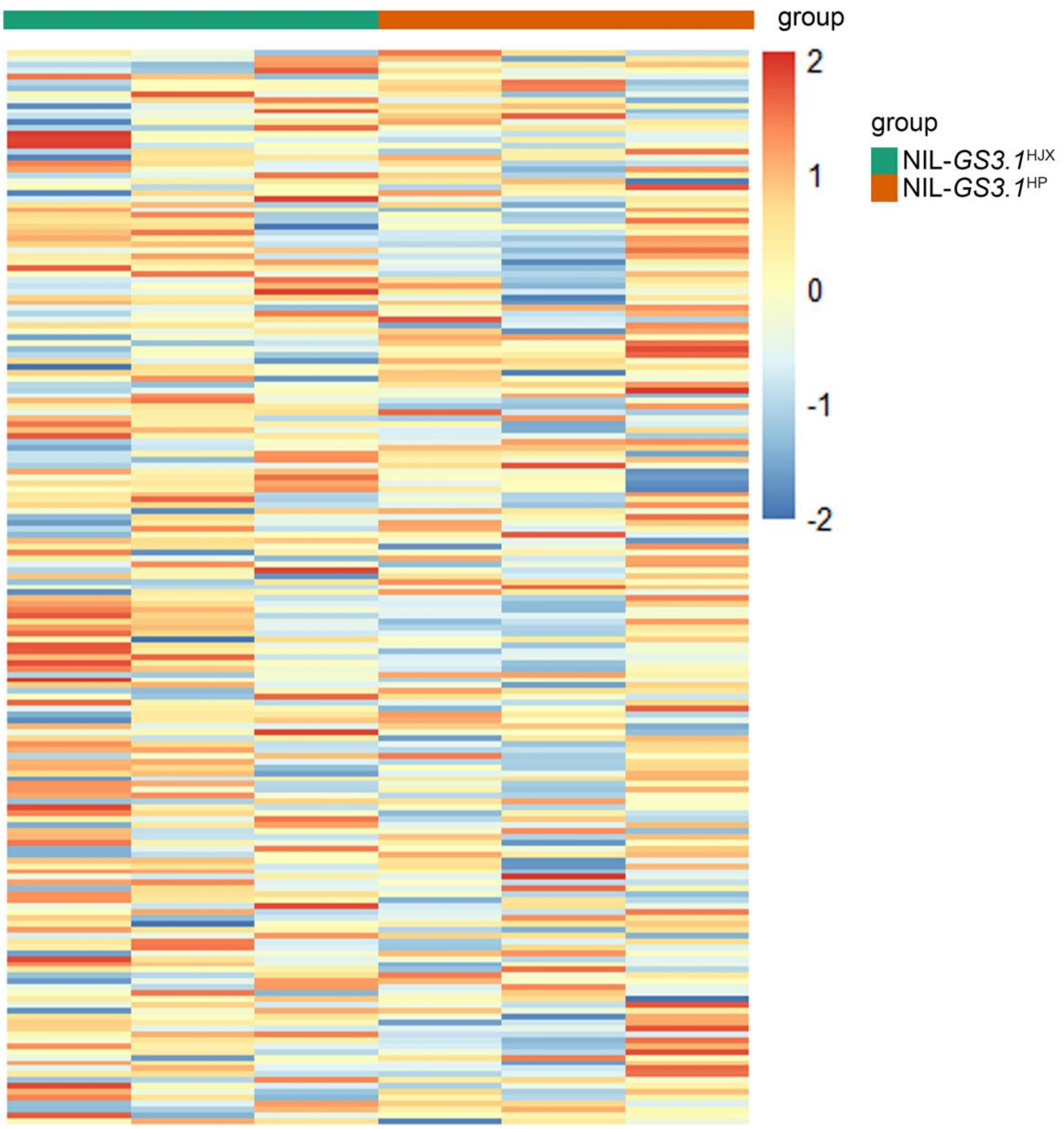

Supplementary Figure 11. Heat maps of the relative content of flavonoids in leaves. Heat maps of the relative content of flavonoids in the leaves of NIL-*GS3.1*^{HJX} and NIL-*GS3.1*^{HP} as determined using metabolomic data. Standard-scores (Z-scores) were used as the numerical metrics to evaluate the standard deviations from the mean of the corresponding samples. The source data underlying Supplementary figure 11 are provided as Source Data file.

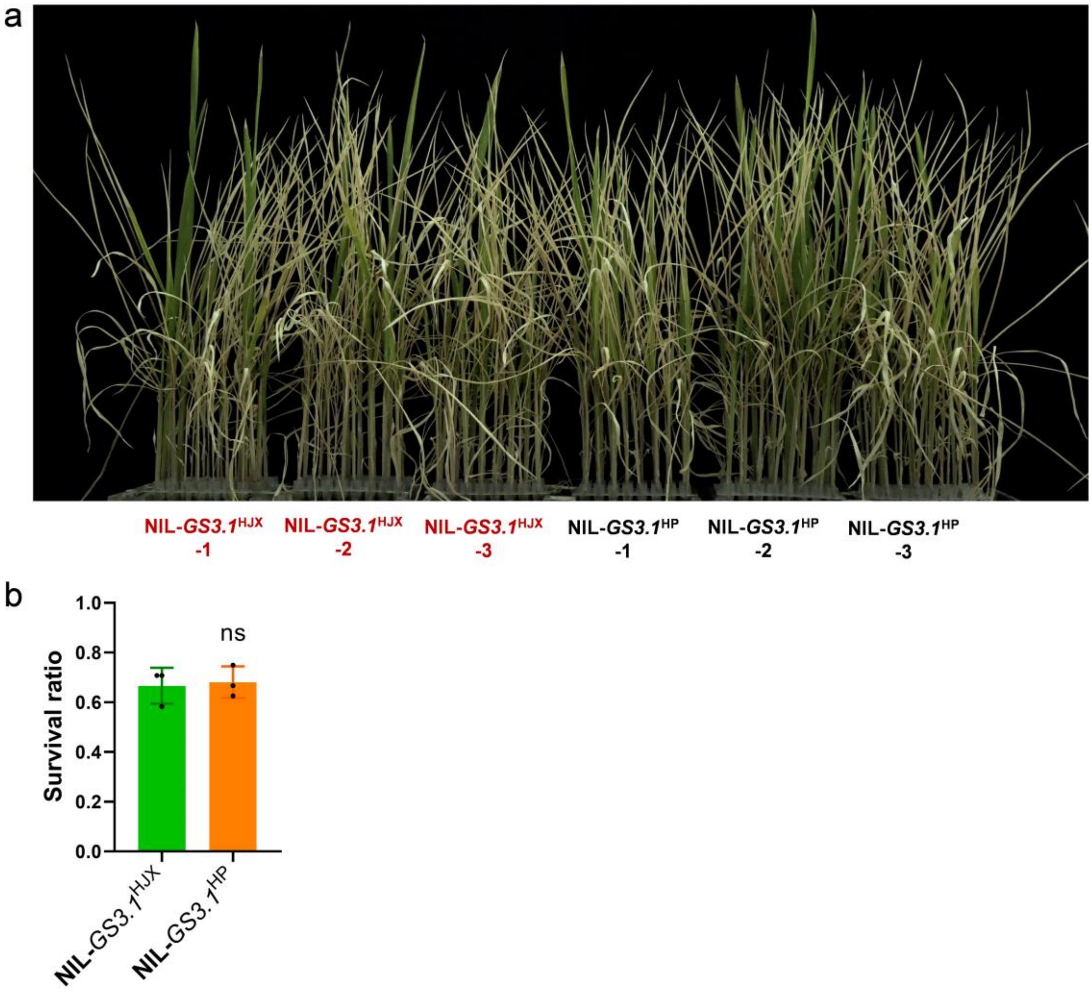

Supplementary Figure 12. There is no difference between NIL-*GS3.1*$^{\mathbf{HP}}$ and NIL-*GS3.1*$^{\mathbf{HJX}}$ in salt tolerance. a, The photograph of the growth status of NIL-*GS3.1*^{HP} and NIL-*GS3.1*^{HJX} seedlings after a 25 days' NaCl treatment and 6 days' recovery. **b**, The survival ratio of the growth status of NIL-*GS3.1*^{HP} and NIL-*GS3.1*^{HJX} seedlings after a 25 days' NaCl treatment and 6 days' recovery. The source data underlying Supplementary Figure 12b are provided as Source Data file.

Supplementary Table 1. *GS3.1* is a QTL contributing to grain size.

	LOD	%VAR	Additive effect	Molecular marker
1000-grain weight	17.514	33.2%	0.608	3-27.208~3-28.502
grain length	11.684	23.6%	0.090	3-27.208~3-28.502
grain width	11.609	23.5%	0.026	3-27.208~3-28.502

Supplementary Table 2. Mutation sites in KO-*GS3.1* lines.

Lines	ko#1	ko#2	ko#6	ko(9522)#4	ko(9522)#11
Mutation sites	Deletion: 58	Insertion (10 bp): 53-54	Deletion: 44-291	Deletion: 205-209	Insertion(1bp): 210-211

Supplementary Table 3. Agronomic phenotypes of NIL-*GS3.1*^{HJX} and NIL-*GS3.1*^{HP}.

	NIL-*GS3.1*^{HJX} (mean±sd)	NIL-*GS3.1*^{HP} (mean±sd)
1000-grain weight (g)	21.65±0.38	23.00±0.42
Grain length (mm)	8.149±0.083	8.304±0.097
Grain width (mm)	2.540±0.018	2.604±0.017
Tiller number	8.067±1.569	8.867±1.454
Spikelet number	274.9±39.2	285.2±46.7
Yield per plant (g)	28.61±7.24	33.38±6.59
Yield per panicle (g)	5.125±0.835	5.713±0.995
Plot yield (g)	2093±74	2275±67

Supplementary Table 4. Cytological data of NIL-*GS3.1*^{HJX} and NIL-*GS3.1*^{HP} outer epidermal cell.

	NIL-*GS3.1*^{HJX} (mean±sd)	NIL-*GS3.1*^{HP} (mean±sd)
Cell number in the grain-length direction	96.67±3.66	96.83±4.09
Cell length (μm)	75.30±6.76	78.73±7.07
Cell number in the grain-length direction	91.6±1.36	91.0±2.45
Cell width (μm)	73.36±8.80	76.82±7.37
