# Supplementary material for: A rice QTL GS3.1 regulates grain size through metabolic-flux distribution between flavonoid and lignin metabolons without affecting stress tolerance
Source: Commun Biol. 2021 Oct 7;4:1171. doi: 10.1038/s42003-021-02686-x (PMC8497587; doi:10.1038/s42003-021-02686-x)
Supplement: Supplementary file 3 — Description of Additional Supplementary Files [file 42003_2021_2686_MOESM3_ESM.pdf]

Description of Additional Supplementary Files

File name: Supplementary Data 1

Description: The source data.

File name: Supplementary Data 2

Description: The primer information.
